# 

**Published:** 1906-07-28

**Authors:** 


					/ HOSPI^AlJ. ^ A "' * * ^
'? ? I1.', f-'N.
July 28xh 1906.
TALr
*sco?r Western Infirmary
j?fica / c?? irtf/nw*nr
^1,086. Vol. XL. Saturday,
July 28th, 1906.

				

